# The low affinity A2B adenosine receptor enhances migratory and invasive capacity *in vitro* and angiogenesis *in vivo* of glioblastoma stem-like cells

**DOI:** 10.3389/fonc.2022.969993

**Published:** 2022-08-18

**Authors:** José I. Erices, Ignacio Niechi, Atenea Uribe-Ojeda, María de los Ángeles Toro, Noemí García-Romero, Josefa Carrión-Navarro, Álvaro Monago-Sánchez, Ángel Ayuso-Sacido, Rody San Martin, Claudia Quezada-Monrás

**Affiliations:** ^1^ Tumor biology laboratory, Institute of Biochemistry and Microbiology, Faculty of Sciences, Universidad Austral de Chile, Valdivia, Chile; ^2^ Millennium Institute on Immunology and Immunotherapy, Universidad Austral de Chile, Valdivia, Chile; ^3^ Faculty of Experimental Sciences, Universidad Francisco de Vitoria, Madrid, Spain; ^4^ Brain Tumour Laboratory, Fundación Vithas, Grupo Hospitales Vithas, Madrid, Spain

**Keywords:** glioblastoma stem-like cells, hypoxia, adenosine, invasion, angiogenesis

## Abstract

Glioblastoma (GBM) is the most common and deadly malignant brain tumor, with a median survival of 15 to 17 months for a patient. GBM contains a cellular subpopulation known as GBM stem-like cells (GSCs) that persist in hypoxic niches and are capable of infiltrating into healthy brain tissue. For this reason, GSCs are considered one of the main culprits for GBM recurrence. A hypoxic microenvironment increases extracellular adenosine levels, activating the low affinity A2B adenosine receptor (A_2B_AR). Adenosine, through A_2B_AR, is capable of modulating invasiveness. However, its role in the invasion/migration of hypoxic-GSCs is still unknown. This study aims to understand the importance of A_2B_AR in modulating the migratory/invasive capacity of GSCs under hypoxia. Data analysis from The Cancer Genome Atlas (TCGA) program correlates A_2B_AR expression with high-grade glioma and hypoxic necrotic areas. U87MG and primary culture-derived GSCs under hypoxic conditions (0.5% O_2_) increased A_2B_AR mRNA and protein levels. As expected, the migratory and invasive capacity of GSCs increased under hypoxia, which was counteracted by blocking A_2B_AR, through the downregulation of MMP9 activity and epithelial–mesenchymal transition marker expression. Finally, in a xenograft mouse model, we demonstrate that treatment with MRS1754 did not affect the tumor volume but could decrease blood vessel formation and VEGF expression. Our results suggest that extracellular adenosine, through the activation of A_2B_AR, enhances the migratory and invasive capacity of GSCs *in vitro* under hypoxic conditions. Targeting A_2B_AR can be an effective therapy for GBM recurrence.

## Introduction

Glioblastoma (GBM) is the most common and aggressive primary brain tumor, accounting for around 54% of all gliomas (1). Current GBM tri-modal treatment includes total surgical resection, radiotherapy, and chemotherapy. However, the survival median is <15 months and only 2% of patients survive for 24 months (2, 3). The poor treatment outcome is mainly caused by the high invasiveness of GBM toward healthy tissue, the difficulty of total removal of malignant cells, and limiting the effects of localized radiotherapy (4). During the past few years, a subpopulation called GBM Stem-like Cells (GSCs) has been identified as being responsible for GBM aggressiveness (5). These cells have stem cell properties (e.g., self-renewal and multi-lineage differentiation), but, unlike neural stem cells, GSCs can initiate the formation and progression of a malignant tumor (6). Different studies have associated GSCs and their high infiltrative capability with tumor relapse, and for this reason, GSCs are becoming more relevant as a target for new treatments for improved patient outcome (6). Tumoral tissue has poorly organized blood vessels, which are unable to correctly provide oxygen, causing hypoxia, which sustains stemness, survival, growth, proliferation, and GSC invasion (7). Additionally, the hypoxic niche of GSCs is composed of endothelial cells (ECs), known as perivascular hypoxia (8). It has been reported that ECs are essential in maintaining the stemness characteristics of GSCs. In fact, studies using co-culture models demonstrated that ECs can induce the expression of genes involved in the stemness phenotype, such as Sox2, Olig2, and Bmil (9, 10). This has also been identified in biopsies from patients diagnosed with GBM, in which it has been reported that CD133+ GSCs reside closely with ECs, indicating the importance of this cellular interaction (11). This hypoxic microenvironment promotes the release of ATP and AMP into the extracellular space, which are hydrolyzed by membrane ectonucleotidases, enhancing adenosine production and allowing cells to persist in the hypoxic niche, thereby promoting stemness-related chemoresistance and invasiveness (12, 13). Previously, we and others have reported that aberrant levels of extracellular adenosine produced by GSCs activate the A_3_ adenosine receptor (A_3_AR) and promote tumorigenic characteristics (14, 15). Interestingly, the A_2B_ adenosine receptor (A_2B_AR) has the lowest affinity for adenosine in comparison to other adenosine receptors, suggesting a relevant role under hypoxic conditions where adenosine is aberrantly increased (A_2B_AR Ki = 15,000 nM *vs* A_2A_AR Ki = 310 nM, A_3_AR Ki = 290 nM, and A_1_AR Ki = 100 nM) (16, 17). Additionally, hypoxia induces A_2B_AR expression in different cancer cells, activating signaling pathways involved in cancer progression (18, 19). Indeed, A_2B_AR downregulation dramatically decreases tumor metastasis in breast cancer, melanoma, and bladder urothelial carcinoma, suggesting an important role of A_2B_AR in cell invasiveness (20). Despite this, the relationship between A_2B_AR and GSC invasion capacity under hypoxic conditions is unknown. Here, we demonstrate that A_2B_AR is highly expressed in high-grade glioma as well as in patient-derived GSCs under hypoxia. Pharmacological blockage of A_2B_AR reduced GSC migration/invasion, expression of EMT markers and MMP-9 activity *in vitro*. In a xenograft model, A_2B_AR blockage GSCs decreased blood vessel formation and angiogenesis markers without affecting tumor volume mass. Our results suggest that extracellular adenosine through the activation of A_2B_AR enhances the migratory and invasive capacity of GSCs under hypoxic conditions *in vitro* and modulates angiogenesis *in vivo*. Targeting A_2B_AR can be an effective therapy for GBM recurrence.

## Material and methods

### Cell culture

The human U87MG GBM (ATCC^®^, HTB-14TM) cell line was grown in differentiation DMEM-F12 medium supplemented with 10% fetal bovine serum and penicillin–streptomycin (Life Technologies, Carlsbad, CA, USA) in standard culture conditions (37°C and 5% CO_2_).

### GBM stem-like cells culture and hypoxia and *in vitro* treatment

For generating GSC from the U87MG cell line (ATCC^®^ HTB-14TM), the cells were grown in neurobasal medium (Gibco, Waltham, MA, USA) supplemented with EGF (20 ng/ml; Peprotech^©^, Rocky Hill, NJ, USA), bFGF (20 ng/ml; Peprotech^©^), 1× B27 (Gibco™), 1× Glutamax (Gibco) and penicillin/streptomycin (100 U/ml, Gibco) at 37°C. After 5 days of culture, GSCs were plated to conduct different tests and treatments. Further, GSC primary cultures (GBM27 and GBM38) were obtained from resected human GBM cultured in M21 medium as described in (21). The work was carried out following the rules of the Declaration of Helsinki. All patients participating in the study gave their informed consent, and protocols were approved by institutional ethical committees. For expansion, GSCs were washed with PBS 1× and then treated for 10 min at 37°C with StemPro^®^ Accutase^®^ (ThermoFisher, Waltham, MA, USA). Subsequently, the cells were maintained in M21 medium for generating new GSC. Normoxia (21% O_2_) and hypoxia (0.5% O_2_) conditions were generated using a gas mixing chamber (5% CO_2_ and 95% N_2_ mixture) for 24 h (18). In *in vitro* studies, MRS1754 (50 nM; Tocris^®^) was used as a selective A_2B_AR antagonist.

### Gliovis analysis

Gene expression and survival data for patients diagnosed with GBM were obtained from records collected by the Cancer Genome Atlas (TCGA) and the Ivy Glioblastoma Atlas, and these were analyzed using the GlioVis web service (Version 0.20) (http://gliovis.bioinfo.cnio.es/, accessed 10 May 2021) (22). Only adult patients whose tumors had mRNA expression obtained by RNA-seq for our genes of interest were included. Pairwise comparisons were performed using the t-test (with Bonferroni correction). The p-values of the pairwise comparisons are indicated on the graphs as ***p <0.001.

### RNA extraction and qRT-PCR cell culture

U87MG and GSCs were maintained under standard culture conditions (37°C, 5% CO_2_) for 4 days. Then, total RNA was extracted using TRIzol Reagent (Thermo Fisher Scientific) and reverse transcription was performed with 1 μg of RNA using the M-MLV Reverse Transcriptase (Thermo Fisher Scientific) following the instructions of the manufacturer. Then, qPCR was performed using the 2^−ΔΔCT^ and β-acting as normalizer genes using HOT FIREPol^®^ EvaGreen^®^ qPCR Mix Plus ROX (Solisbiodine) following the instructions of the manufacturer. The qPCR reactions were performed with 250 nM of each primer (**Supplementary Table 1**).

### Western blot

Total proteins extracts (40 μg) obtained from U87MG GSCs and PC GSCs were fractionated by SDS-PAGE, transferred to 0.22 μm PVDF membranes (General Electric, GE^®^, Boston, MA, USA) and blocked with 1× PBS/0.05% Tween/1% BSA or 5% non-fat milk for 1 h. Then, membranes were incubated overnight with primary antibodies at 4°C followed by a secondary antibody-HRP conjugate during 1 h. Western blots were revealed using the SuperSignal™ West Dura Extended Duration Substrate kit (Thermo Fisher Scientific) and images were quantified by densitometry analysis (ImageJ, NIH). Primary antibodies targeting the following proteins were used: A_2B_AR (Abcam, cat#ab222901; 1:1,000); Mmp9 (Cell Signaling, cat#13667, 1:1,000); β-actin (Santa Cruz, cat#sc-47778, 1:5,000).

### Cell adhesion assay

Pre-treated GSCs were seeded in a 96-well plate (2 × 10^4^ cells/well) and incubated at 37°C for 2 h in neurobasal medium plus 1% FBS to trigger cell adhesion (23). Non-adherent GSCs were carefully removed by inverting the culture plate, and adherent GSCs were fixed (PFA 3.7% for 10 min) and stained (0.5% crystal violet/20% methanol for 10 min). GSCs were washed with 1× PBS and incubated with 10% acetic acid (v/v) for 5 min. Cell adhesion was analyzed by measuring the optical density of the eluted crystal violet stain at 570 nm in a microplate reader (Synergy 2 Biotek).

### Migration and invasion assay

GSCs were plated (75,000 cells/chamber) on the top side of a polycarbonate Transwell chamber (6.5 mm, 8.0 μm pore Polycarbonate membrane; Corning) for migration assay or in a 300 µg/ml matrigel-coated Transwell chamber (Corning) for invasion assay. Cells were seeded in serum-free neurobasal medium for U87 or M21 for primary cultures. As a chemoattractant medium, 10% FBS DMEM-F12 was used in the bottom chamber. Cells were incubated at 37°C for either 6 h or 12 h for migration or invasion assays, respectively. The cells were stained for 15 min with 1% crystal violet/20% methanol, after they were washed with water, and the sediment in the top chamber was carefully removed with cotton swabs. Cells were counted using ×10 objectives in five different fields on the underside of the insert. The mean number of cells was normalized to 1 using the control conditions and then plotted.

### Gelatinase activity

A Biovision Gelatinase Fluorogenic Activity Assay kit (Millipore Sigma, Burlington, MA, USA) was used to assess the activity of gelatinase in cells. This assay uses a triple-helical collagen-like peptide that is highly selective for MMP2 and MMP9. This approach relies on the fluorescence resonance energy transfer (FRET) phenomenon. The fluorescence of the FRET peptide is quenched while it is intact and released only when the peptide is cleaved. Accordingly, this peptide is used as an indicator of MMP activity. The manufacturer protocol was followed. Finally, fluorescence was measured *via* a microplate reader (Synergy 2 Biotek) with excitation and emission wavelength of 320 and 405 nm, respectively.

### Heterotopic xenograft assay and MRS1754 treatment

Resuspended in culture media mixed with cold liquid Matrigel (BD) were 1.5 × 10^6^ GBM38 cells, and they were subcutaneously injected into athymic nude mice (N = five mice per group). Tumor volumes were measured every two days with a caliper and treatment with MRS1754 started when the tumors reached >25 mm^3^, administering 160 ng per kg every 48 h. Mice were euthanized 20 days after the beginning of treatment, before the tumor burden became symptomatic.

### RT-qPCR from tumor tissue samples

For qRT–PCR, total RNA from tumor samples was isolated by phenol–chloroform extraction. One microgram of isolated RNA was used for cDNA synthesis (High-Capacity cDNA Reverse Transcription Kit; Applied BioSystems). QRT–PCR reactions were performed in an optical 96-well plate equipped with a Bio-Rad CFX Connected Real-Time PCR Detection System, using SYBR Green. Gene expression levels were quantified using the primers listed in **Supplementary Table 1**, while the β-actin housekeeping gene was used to normalize the data.

### Immunofluorescence staining and quantification

Tumor pieces were fixed with 4% PFA, embedded in paraffin and cut into 3 µm sections. Tumor sections were immersed in a 10% blocking solution of the specific serum and then incubated (overnight, 4°C) in solutions containing the following primary antibodies: goat anti-mouse CD105 (R&D Systems, ref. AF1320), mouse anti-human vimentin (Santa Cruz Biotechnology, ref. sc-6260) and rabbit anti-VEGF (Abcam, ref. ab52917). Then, Alexa Fluor-conjugated secondary antibodies were used for 1 h (donkey anti-goat 568 and goat anti-mouse 660, LifeTechnologies, ref. A-11057 and A-21055, respectively; and donkey anti-rabbit 647, Abcam ref. ab150075), and the nuclei were counterstained with DAPI. Coverslips were mounted using Fluor-Save™ reagent (Calcibiochem, ref. 345789). Five random areas of each tumor were imaged using a Leica Microsystems THUNDER 3D Assay Imager epifluorescence microscope. Immunofluorescence images were quantified with the open-source software FiJi version 2.3.051. The background was subtracted from CD105 and VEGFA raw intensity images, then the integrated intensity density of each image was multiplied by the number of pixels in the image and divided by the number of cells that were stained by DAPI.

### Statistical analysis

GraphPad Prism^®^ 8.01 software was used to perform the statistical analysis. Values are shown as mean ± S.D., where n indicates the number of independent experiments. A Student’s t-test was applied for unpaired data. P- and P-adjusted values ≤0.05 were considered statistically significant. For *in vivo* analysis, data were assessed for normality with either the Shapiro–Wilk or Kolmogorov–Smirnoff tests depending on the number of samples. Group comparison was performed with an unpaired, two-tailed T-test for data that passed the normality test or by the Mann–Whitney U-test for data that were not deemed normal. For both cases, p-values are represented as follows: ***p <0.001, **p <0.01, *p <0.05; differences among groups were not considered statistically significant when p >0.05.

## Results

### The expression of A2B adenosine receptor is associated with high glioma grade and lower patient survival

**Figure 1:**
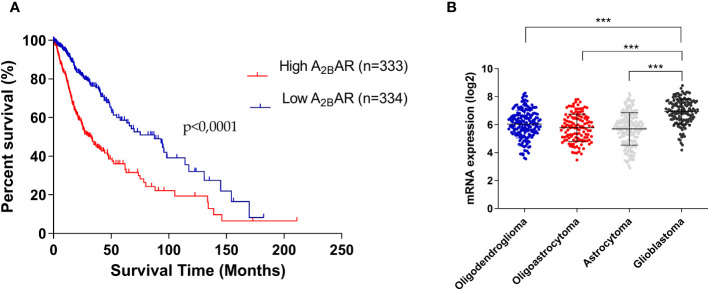
Overexpression of A_2B_AR is associated with worse GBM prognostis. **(A)** Kaplan–Meier analysis of the association between A_2B_AR expression and glioma patient survival (Median survival: low A_2B_AR expression 87.50 months *vs* high A_2B_AR expression 32.50 months. Cut-off used was the median of all samples (6.5722). p-value = 0.0001; n = 667). **(B)** A_2B_AR expression according to glioma grade (oligodendroglioma (n = 189), oligoastrocytoma (n = 126), astrocytoma (n = 191), and glioblastoma (n = 149). Statistical significance was determined by one-way ANOVA, ***p-value <0.001.

A_2B_AR influence in glioma-patient survival was studied using data from GlioVis (including Oligodendroglioma, Oligoastrocytoma, Astrocytoma, and GBM). We performed Kaplan–Meier curves to explore correlations between A_2B_AR expression and patient survival (**Figure 1A**). Patients with high A_2B_AR levels have shorter overall survival than those who have low A_2B_AR levels (Median Survival for Low A_2B_AR 87.50 months *vs* High A_2B_AR 32.50 months *vs* Low High (p-value = 0.001). Finally, A_2B_AR levels increase with glioma tumor grade, with overexpression in GBM tissue (**Figure 1B**). These results suggest that high A_2B_AR expression might be associated with progressive malignancy in gliomas and is specific to GBM.

### A_2B_ adenosine receptor upregulation under hypoxic GSCs promotes *in vitro* invasiveness

**Figure 2:**
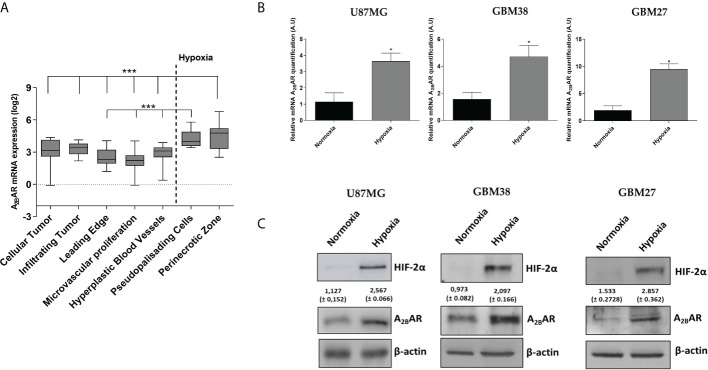
A_2B_AR expression increases under hypoxia conditions. **(A)** mRNA expression data were retrieved from the IVY Glioblastoma project and analysed for A_2B_AR expression (n = 55 samples). A_2B_AR mRNA was increased in the hypoxic pseudopalisades and peri-necrotic areas of glioblastoma (GBM) samples. Statistical significance was calculated by one-way ANOVA. (***P < 0,001). **(B)** A_2B_AR mRNA levels were analyzed in three GSC lines (U87MG, GBM38, and GBM27) under normoxia and hypoxia conditions for 24 h. Values were normalized to β-actin mRNA expression (*p-value <0.05; n = 3). Y-axis values are represented in arbitrary units (A.U). **(C)** Representative Western blot of HIF-2α, A_2B_AR and β-actin of the three GSC lines under normoxia and hypoxia for 24 h.

Searching for A_2B_AR expression sites in GBM tissue according to the Ivy database, we found that A_2B_AR was predominantly expressed in GBM tissues located in invasion-related hypoxic regions such as the pseudopalisades and perinecrotic areas (24) (**Figure 2A**). To confirm this, we evaluated mRNA and protein levels in GSCs obtained from three different cell types cultured under hypoxia: U87MG and two primary cultures (GBM38 and GBM27). A_2B_AR mRNA (**Figure 2B**) and protein (**Figure 2C**) levels increased under hypoxia, suggesting its importance in GSC maintenance under low O_2_ pressure.

**Figure 3:**
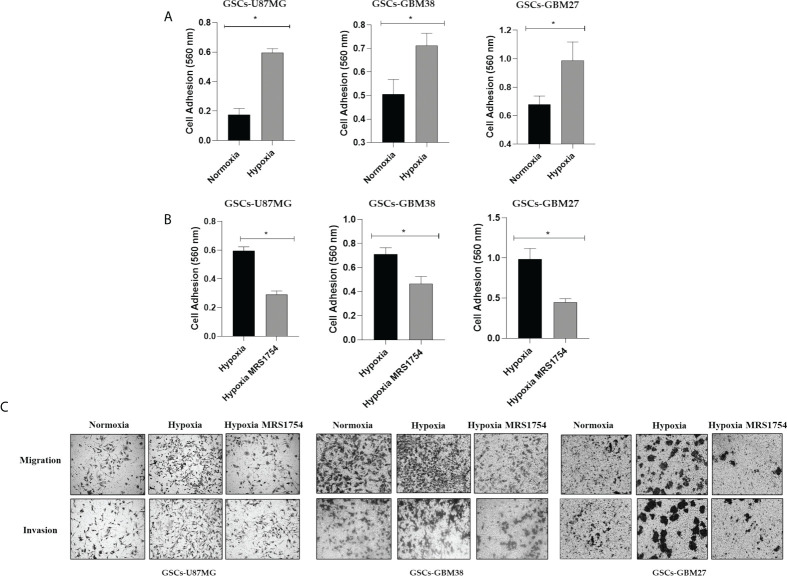
Hypoxia induces adhesion, migration, and invasion of GSCs. **(A)** Cell adhesion at 2 h in U87MG, GBM27, and GBM38 under normoxia (black box) and hypoxia (gray boxes) pre-incubated under hypoxia for 24 h. **(B)** Cell adhesion at 2 h in U87MG, GBM27, and GBM38 under hypoxia (black box) and hypoxia MRS1754 (gray boxes) pre-incubated under hypoxia for 24 h. Statistical significance was determined by Student t-test, *p-value <0.05. **(C)** Migration and Invasion (transwell and transwell matrigel-coated assay, respectively) in U87MG, GBM38, and GBM27 under hypoxia for 24 h.

**Figure 4:**
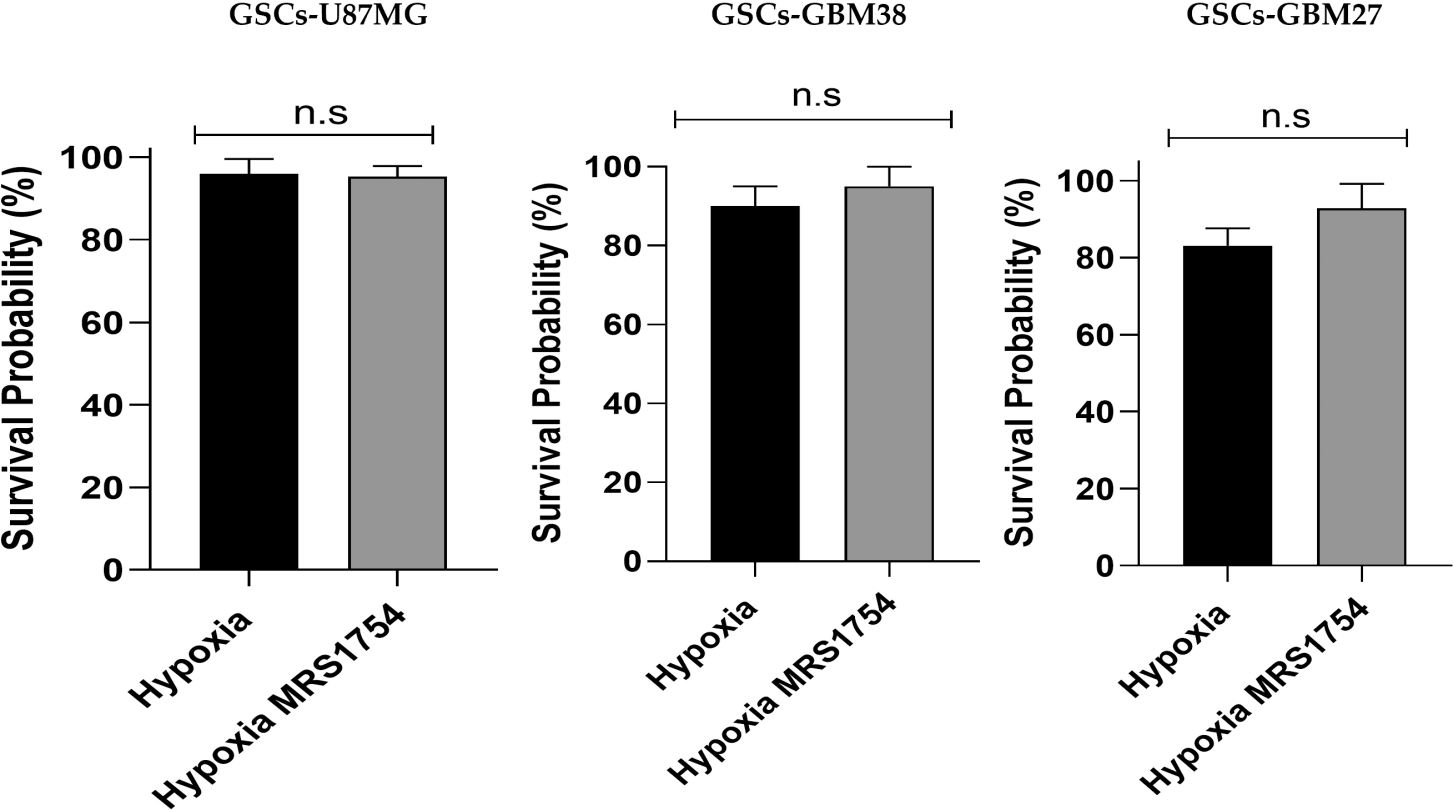
Effect of A_2B_AR pharmacological blockade on cell viability of glioblastoma stem-like cells under hypoxic conditions. The cell viability of U87MG, GBM38, and GBM27 was determined through the MTS assay. Ten thousand cells from each cell culture were seeded and cultured in the experimental conditions under which the transwell and transwell-matrigel assays were performed. The absorbance of the MTS was read at 490 nm on an ELISA plate reader, with values expressed as percentages. The graphs represent the mean ± SD. n.s., non-significant.

Hypoxia has been identified as responsible for high GBM recurrence by enhancing infiltration into healthy brain tissue (25). For this reason, we evaluated the impact of hypoxia on GSC adhesion, migration, and cell invasion. To analyze cell adhesion, we performed a fibronectin-coated plate assay with U87MG, GBM38, and GBM27, demonstrating that hypoxia induced *in vitro* cell adherence of all cells (**Figure 3A**). To assess migration and invasion capacities, transwell and transwell-matrigel assays were performed, respectively. As expected, migration and invasion of GSCs increased under hypoxia (**Figure 3C**). These results confirm that hypoxia, which is related to A_2B_AR overexpression, is also related to the GSC infiltrative phenotype. To further investigate the role of A_2B_AR signaling in GSCs, we used MRS1754 (50 nM), a selective A_2B_AR-antagonist (26). A_2B_AR signaling blockage under hypoxic conditions impaired cell adhesion, migration, and invasion of U87MG, GBM38, and GBM27 (**Figures 3B, C** **)**. To rule out a cytotoxic effect of MRS1754 on GBM cells, we performed an MTS assay. We found that MRS1754 had no effect on GSC viability (**Figure 4**). All together, these results suggest that A_2B_AR signaling is a suitable target for decrease GSC hypoxia-related invasion.

### A_2B_AR blockage decreases endothelial-mesenchymal transition markers and MMP9 activity *in vitro*

**Figure 5:**
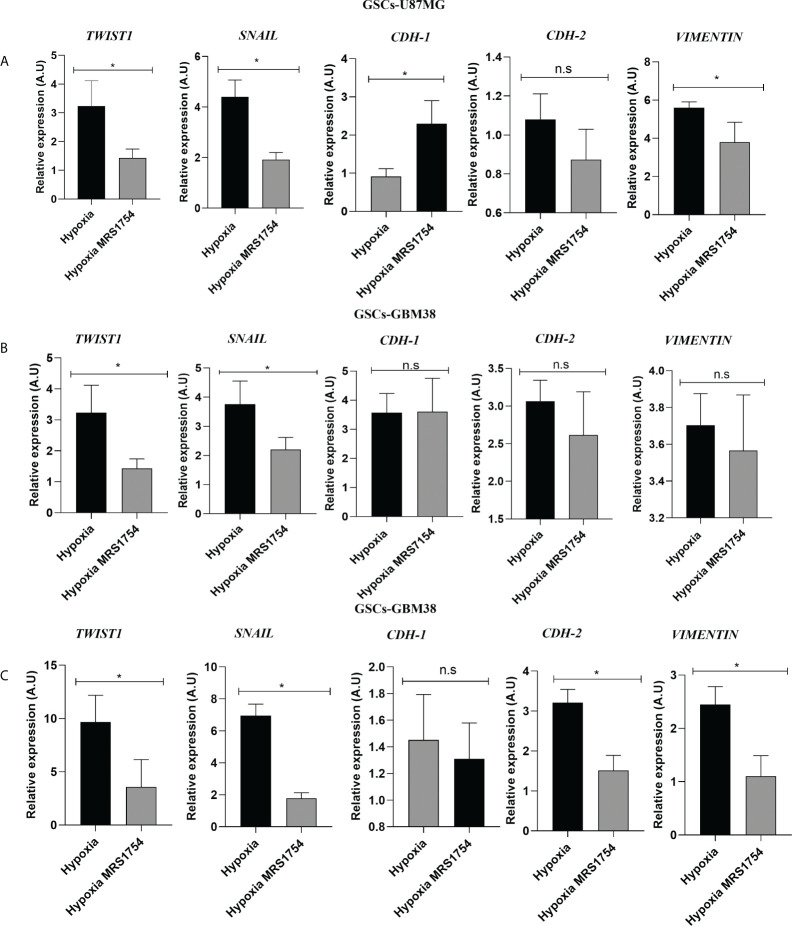
The A2B Adenosine Receptor controls the expression of Epithelial–Mesenchymal Transition markers in Glioblastoma Stem-like Cells under hypoxia. RT-qPCR of *TWIST1*, *SNAIL*, *CDH-1*, *CDH-2*, and *VIMENTIN* in GSCs derivated from **(A)** U87MG, **(B)** GBM27, and **(C)** GBM38 under hypoxia and hypoxia MRS1754 from 24 h. Values were normalized to β-actin mRNA expression. Y-axis values are represented in arbitrary units (A.U). Statistical significance was determined by the Student t-test; *p-value <0.05; n = 3.; n.s., non-significant.

**Figure 6:**
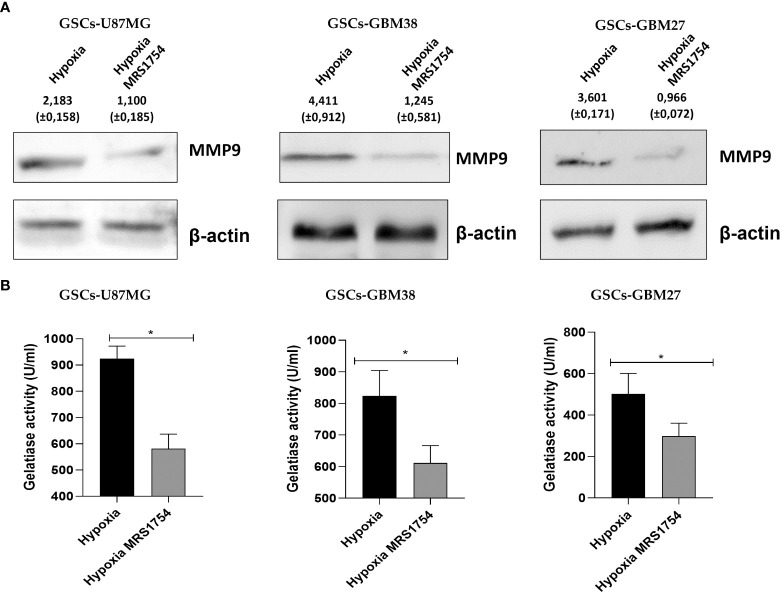
MRS1754 reduces MMP9 protein levels and gelatinase activity in GSCs treated with MRS1754 under hypoxia conditions. **(A)** Immunoblot of MMP-9 from total lysate of U87MG, GBM27, and GBM38 under hypoxia and hypoxia MRS1754. β-actin was used as a loading control. **(B)** Quantification of gelatinase activity from U87MG, GBM27, and GBM38 incubated under hypoxia and hypoxia MRS1754. The assays were performed according to the kit protocol. Statistical significance was determined by the Student t-test, *p <0.05; n = 3.

Invasiveness progression is associated with endothelial–mesenchymal transition (EMT) in different types of cancers, including GBM (27, 28). To study the role of A_2B_AR signaling on EMT, we evaluated mRNA expression of *SNAIL*, *TWIST1*, *CDH-1*, *CDH-2*, and *VIMENTIN* in all cell lines grown under hypoxia and treated with MRS1754. A_2B_AR blockage reduced mRNA levels of *TWIST1* and *SNAIL* in all GSCs under hypoxic conditions and decreased *VIMENTIN* levels and increased *CDH-1* in GSCs-U87MG. Finally, *TWIST1* and *SNAIL* mRNA levels were downregulated under A_2B_AR blockage in hypoxic GSCs-GBM38 and GSCs-GBM27 **(** **Figure 5** **)**. Matrix-metalloproteinase-9 (MMP-9) is relevant to GBM infiltration. As expected, A_2B_AR blockage decreased MMP9 protein expression (**Figure 6A**), which was related to downregulation of gelatinase activity (**Figure 6B**) under hypoxic conditions in all GSCs. All together, these results suggest that the invasion/migration of GBM cells through EMT gene expression and MMP9 activity is A_2B_AR-dependent under hypoxic conditions.

### A_2B_AR blockage reduces *in vivo* GBM angiogenesis expression marker

**Figure 7:**
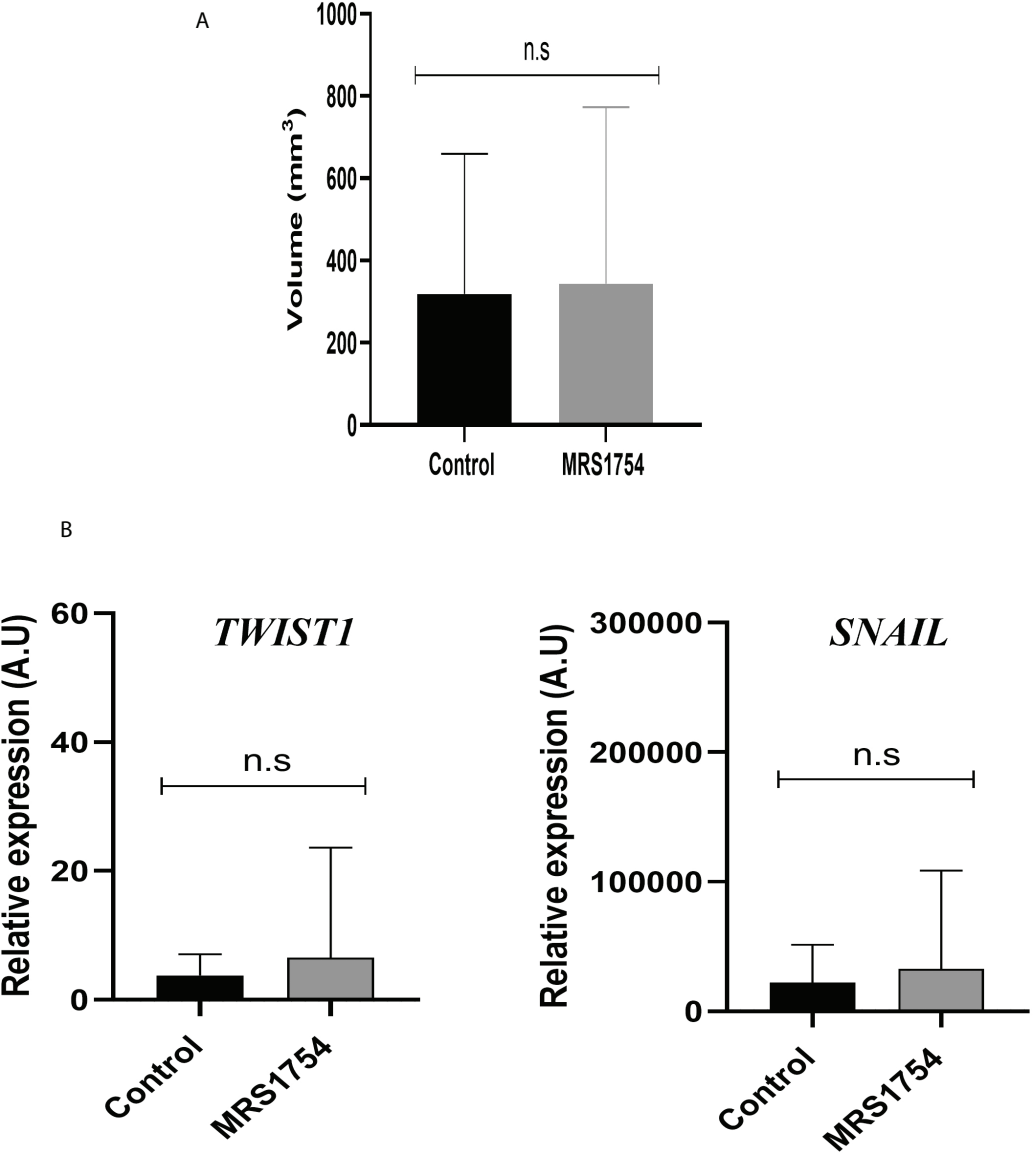
MRS1754 does not reduce the tumor volume and EMT marker expression *in vivo* on subcutaneous GBM38. Tumors were generated by subcutaneously implanting GBM38 GSCs in nude mice. **(A)** Graph of tumour size (mm^3^) of *in vivo* treatment. Treatments started when the tumour reached a size of >25 mm^3^, injecting 1× PBS-0.001% DMSO (Vehicle) or MRS1754 160 ng/kg/48 h in 0.01% DMSO (treatment).n.s, non-significant. **(B)** RT-qPCR of *TWIST1* and *SNAIL1* from tumor tissue. Values were normalized to β-actin mRNA expression.

**Figure 8:**
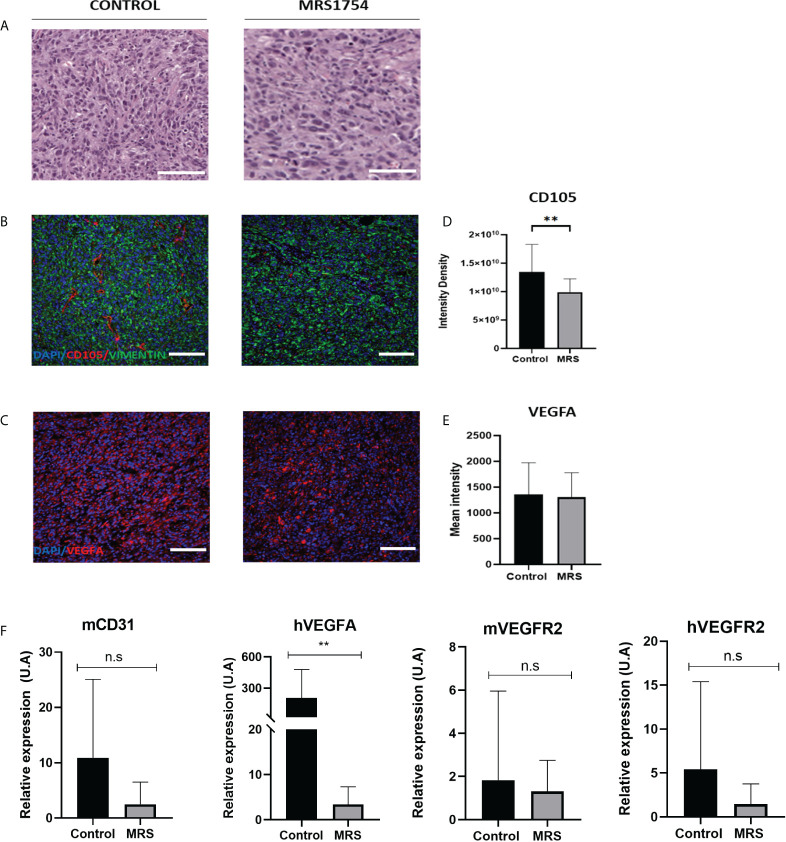
MRS1754 reduces tumor blood vessel formation and angiogenesis marker expression *in vivo* on GBM38. **(A)** H&E staining of representative sections of tumors from control and MRS treatment groups. Scale bar is 200 µm. **(B)** Immunofluorescence images of representative areas from control and MRS treatment groups. Stained with DAPI (in blue), CD105 (in red), and Vimentin (in green). The scale bar is 100 µm. **(C)** Immunofluorescence images of representative areas from control and MRS treatment groups. Stained with DAPI (in blue) and VEGFA (in red). The scale bar is 100 µm. **(D)** Quantification of CD105 levels in immunofluorescence images. Shown here is the integrated intensity density of CD105 in the control and MRS treated groups. **p value <0.01. **(E)** Quantification of VEGFA levels in immunofluorescence images. Shown here is the mean intensity of VEGFA in the control and MRS treated groupsis shown here. **(F)** mRNA expression levels of mCD31, hVEGFA, hVEGFR2, and mVEGFR2 in both control and MRS treatment groups. **p value <0.01. n.s, non-significant.

Finally, we evaluated the blockage of A_2B_AR by MRS1754 in a heterotopic xenograft model subcutaneously injected with GBM38-derived GSCs. Like proliferation *in vitro* results, we did not observe any differences in tumor volume mass (**Supplementary Figure 2**) (**Figure 7A**). As we observed differences in the expression of *TWIST1* and *SNAIL* mRNA levels in GBM38s *in vitro*, we evaluated the expression of these same genes in our *in vivo* model. In contrast to our previous results, we did not observe differences in mRNA expression levels of *TWIST1* and *SNAIL* n the tumoral tissue treated with MRS1754 (**Figure 7B**). Despite this, due to the importance of A_2B_AR in the regulation of angiogenesis, which is strongly related to tumor maintenance and recurrence, we evaluated the expression of angiogenesis markers by immunofluorescence. Interestingly, CD105^+^ [Endoglin, associated with microvascular proliferation in GBM (29)] blood vessel formation and VEGFA marker decreased under A_2B_AR blockage *in vivo* (**Figures 8A–E**). Also, we observed a decrease in *VEGFA* mRNA levels, along with a lower expression of *VEGFR* in humans and mice (**Figure 8F**). All together, these results suggest that adenosine through A_2B_AR is involved in the regulation of angiogenesis in GBM tumors *in vivo.*

## Discussion

In the last few years, a subpopulation called GSCs has been identified as being responsible for the aggressiveness of GBM. These GSCs present stem-cell properties like self-renewal and multi-lineage differentiation, but GSCs can initiate the formation of the tumour. Different studies have implicated these GSCs in the relapse of GBM patients due to their high infiltrative capability (30). GSCs reside in a specific tumor microenvironment (TME), which promotes the maintenance of this type of cell. We previously reported that extracellular adenosine levels increased in GSCs when under hypoxia conditions (14). Adenosine can activate A_1_, A_2A_, and A_3_ receptors, which are classified as high affinity receptors that activate at mM adenosine concentrations, but it has been reported that A_2B_AR is only a low affinity receptor (12), which is activated by high extracellular adenosine levels observed under hypoxia (31, 32). Despite the importance of A_2B_AR signaling on cell adaptation to low oxygenation levels, the role of A_2B_AR on GSC invasiveness under hypoxia has not been described. Our analyses showed A_2B_AR levels correlated with shorter survival times in patients, and that A_2B_AR was overexpressed in GBM, especially in hypoxic regions like pseudopalisades and the perinecrotic zone, which have been associated with a high invasive potential of the GBM cells (33, 34). To validate this analysis, we incubated GSCs derived from three different GBM cells under hypoxic (0.5% O_2_) conditions. We observed that mRNA and protein levels of A_2B_AR increased in GSCs cultured under hypoxic conditions. These results are consistent with those previously described by other authors, which indicate that the expression of A_2B_AR is associated with stressful and physio-pathological conditions such as low oxygen tension, which induces an increase in adenosine levels (35). As hypoxic niches have been considered a poor prognostic marker for GBM (36), it has been demonstrated that cells found in areas with low oxygenation increase their invasiveness by 60% compared to cells located in areas of higher oxygenation (37), and similar results were observed in our experimental assay. In this work, we show that pharmacological blockade of A_2B_AR significantly reduces the adhesion of GSCs, suggesting that the signaling of this receptor could have an impact on the regulation of integrins. In fact, there is some evidence suggesting that adenosine can regulate the expression and localization of this type of surface protein in different cellular models (38). Yi et al. showed MRS1754 reduces migration and invasion of renal carcinoma (39). Meanwhile, increasing evidence suggests Ecto-5’-nucleotidase (CD73) derived adenosine also enhances tumor cell migration *via* A_2B_R (40). Also, the expression of A_2B_AR in a metastatic colorectal cancer cell line (SW620) is significantly higher than in a non-metastatic model (SW480) (41), showing the regulatory role of A_2B_AR in cancer cell invasion.

In GBM, despite being a non-epithelial neoplasia, there is clear evidence that cells comprising that type of malignant brain tumor are capable of an EMT-like process (27, 28). Mikheev et al. evaluated the gene expression of 85 biopsies derived from patients diagnosed with GBM, where the transcription factor Twist, which regulates the expression of genes involved in EMT, was overexpressed compared to healthy tissue sections, demonstrating the importance of this transcription factor as a modulator of the cell invasion process in GBM (42). It has been previously described that adenosine may participate in promoting GBM invasion (14, 43, 44). However, the role of A_2B_AR signaling on EMT marker expression has not yet been described. These results indicate that A_2B_AR can regulate the expression of Twist1 and Snail, but we can appreciate different impacts on other EMT markers like *CDH-1* and *CDH-2*. Also, A_2B_AR blockage decreases the expression and activity of MMP-9, which is important for GBM infiltration of non-GSCs GBM cells into healthy brain tissue (45). This evidence and our results suggest a role for A_2B_AR in enhancing the infiltrative phenotype of GBM and GSCs. Although this is the first evidence of the role of A_2B_AR in the EMT process in GSCs, the role of this receptor in the regulation of EMT has been previously reported. It has been previously described that A_2B_AR activation decreased the expression of epithelial markers (E-cadherin) and enhanced mesenchymal markers (Vimentin, N-cadherin) during pulmonary fibrosis (46). In the case of GSCs, our research group has already demonstrated that adenosine can regulate the expression of some EMT markers, impacting on the invasive capacity of GSCs, specifically through A_3_AR (14). In the same way, it has been described that adenosine, through A_3_AR activation, regulates the EMT process in head and neck cancers because the blockade of this adenosine receptor decreased the expression of proteins such as Vimentin, Zeb, Slug, and Twist1, which are essential during the EMT process and the invasiveness of this type of neoplasia (47). A similar effect was reported by Tsiampali et al. in which they described that adenosine production by Ecto-5’-nucleotidase/CD73 was capable of activating EMT through increased expression of Snail and ZEB1 *via* the activation of A_3_AR (48). The latter study only focused on differentiated cells, but nevertheless, it provides evidence for the importance of adenosine signalling in EMT.

Despite the heterogeneity presented by GBM cells, pharmacological blockage of A_2B_AR decreased invasiveness in all three cell lines under hypoxic conditions. Clear evidence exists that hypoxia can provoke a molecular shift in GSCs toward the mesenchymal (MES) subtype. Bao et al. observed that GBM cell lines that do not have mesenchymal characteristics, when cultured under hypoxia, induce the expression of the MES gene within the higher invasive capacity (49). It is possible to suggest that hypoxia, through A_2B_AR, induces an invasive phenotypic change, such as the mesenchymal type. In fact, the increased expression of A_2B_AR was related to the MES subtype of GBM (**Supplementary Figure 2**).

Although we did not observe differences in the expression of EMT markers, we observed that tumors treated with MRS1754 exhibited a reduced formation of blood vessels, in addition to a lower expression of VEGF and VEGFR. Endothelial cells express high levels of A_2B_AR, suggesting its potentially critical role in promoting angiogenesis (50). Du et al. showed that the activation of A_2B_AR by NECA significantly increased the intracellular cAMP levels and concomitant CREB phosphorylation, eventually leading to the production of VEGF in HMEC-1 (50). Also, in the tumoral microenvironment, A_2B_AR activation is involved in the synthesis of VEGF and IL-8 in endothelial cells and stromal tumor cells (51). In melanoma tissues, A_2B_AR receptor activation induces an increase in VEGF by activating the transcription factor STAT3 (52). In a GBM cell line derived from a mouse line expressing A_2B_AR, this receptor is highly upregulated, leading to angiogenesis (52).

Our results suggest an eventual therapy focused on adenosine signaling, mainly through blocking A_2B_AR, since its low affinity for adenosine postulates it as a potential target for pathological conditions, such as cancer (52). In addition to our observations, the blockade of A_2B_AR signaling significantly decreases the invasive and angiogenic potential of GSCs, it making it an attractive therapeutic target to decrease the high recurrence of GBM. However, we must emphasize that our study has some limitations, mainly focusing on our subcutaneous models because they do not represent the tumor context of GBM. For this reason, it is of utmost importance to use GBM models such as the intracranial mouse model or the use of organoid models (53, 54). This will allow us to have a more complete view of the role of A_2B_AR on the invasiveness of GSCs and to design a more effective treatment. As a projection, we propose an eventual therapy targeting the extracellular adenosine axis, which would not only affect the cells that surround the tumor but also GSCs in the hypoxic niches that are responsible for the progression and recurrence of GBM.

## Data availability statement

The raw data supporting the conclusions of this article will be made available by the authors, without undue reservation.

## Ethics statement

The animal study was reviewed and approved by the ANID.

## Author contributions

JE conceived the project and designed all the experiments. JE, AU-O, and MT carried out most *in vitro* experiment. NR, JN, AMS, and AAS carried out the *in vivo* experiment. JE, IN, CQ, RS, and NR wrote and revised the manuscript. Data analyses were performed by JE, AU-O, MT, IN, and CQ. All authors listed have made a substantial, direct, and intellectual contribution to the work and approved it for publication.

## Funding

The study was supported by the Fondecyt regular 1200885, (CQ-M) Agencia Nacional de Investigació́n y Desarrollo (ANID)— Millennium Science Initiative Program—ICN09_016/ICN 2021_045: Millennium Institute on Immunology and Immunotherapy (ICN09_016/ICN 2021_045; former P09/016- F), (CQ-M), Vicerrectoría de Investigación, Desarrollo y Creación artistica VIDCA-UACh and the CONICYT Postgraduate scholarship N° 21181983 (JIE). Also, this work was supported in part by grants from the “Fondo de Investigaciones Sanitarias” (FIS) (PI21/01353) and the Ministerio de Economía y Competitividad–FEDERER (RTC-2019-6918-1)(AA-S).

## Conflict of interest

The authors declare that the research was conducted in the absence of any commercial or financial relationships that could be construed as a potential conflict of interest.

## Publisher’s note

All claims expressed in this article are solely those of the authors and do not necessarily represent those of their affiliated organizations, or those of the publisher, the editors and the reviewers. Any product that may be evaluated in this article, or claim that may be made by its manufacturer, is not guaranteed or endorsed by the publisher.
