# Supplementary material for: The low affinity A2B adenosine receptor enhances migratory and invasive capacity in vitro and angiogenesis in vivo of glioblastoma stem-like cells
Source: Front Oncol. 2022 Aug 18;12:969993. doi: 10.3389/fonc.2022.969993 (PMC9433907; doi:10.3389/fonc.2022.969993)
Supplement: Supplementary file 1 [file DataSheet_1.docx]

Supplementary Material

**Table 1**. Primer sequences used for *in vitro* and *in vivo* assays.

| ***Gene*** | ***Forward sequence*** | ***Reverse sequence*** |
| --- | --- | --- |
| mCD31 | CGGTTTCCTAAGGTCTGAGC | GAGAAGGCGAGGAGGGTTAG |
| hVEGFA | CTAACACTCAGCTCTGCCC | ACACACAAATACAAGTTGCCAA |
| mVEGFA | ACACGGTGGTGGAAGAAGAG | GGAAGGGAAGATGAGGAAGG |
| hVEGFR2 | GCTCGCCTCCCTTTGAAAT | ATTTCCCACAGCAAAACACC |
| mVEGFR2 | GGCAGTGTCTGAGGGTTCTC | TGGAGAGCAAACCAACCAAT |
| hVimentin | GTCTTGACCTTGAACGCAAAGTGG | GGACATGCTGTTCCTGAATCTGAG |
| hE-cadherin | GAGGAATCCAAAGCCTCAGGTCAT | TCACCCACCTCTAAGGCCATCTTT |
| hN-cadherin | TCCTATGAGTGGAACAGGAACGCT | AATTGGGGTCTGGAGTTTCGCA |
| hSnail1 | CTTCTCACTGCCATGGAATTCCCT | TCCACAGAAATGGCCATGGGAAA |
| hTwist | TCAGCCACTGAAAGGAAAGGCA | GCAGGCCAGTTTGATCCCAGTATT |
| hMMP9 | ATTTCTGCCAGGACCGCTTCTACT | TGTCATAGGTCACGTAGCCCACTT |
| hHIF1α | TGCTGACCCTGCACTCAATCAA | TTCCATCGGAAGGACTAGGTGTCT |
| hHIF2α | GACAAGGTCTGCAAAGGGTTTTGG | GGAAGGCTTGCTCTTCATACTCCA |
| hβ-Actin | TTCTACAATGAGCTGCGTGTG | GGGGTGTTGAAGGTCTCAAA |


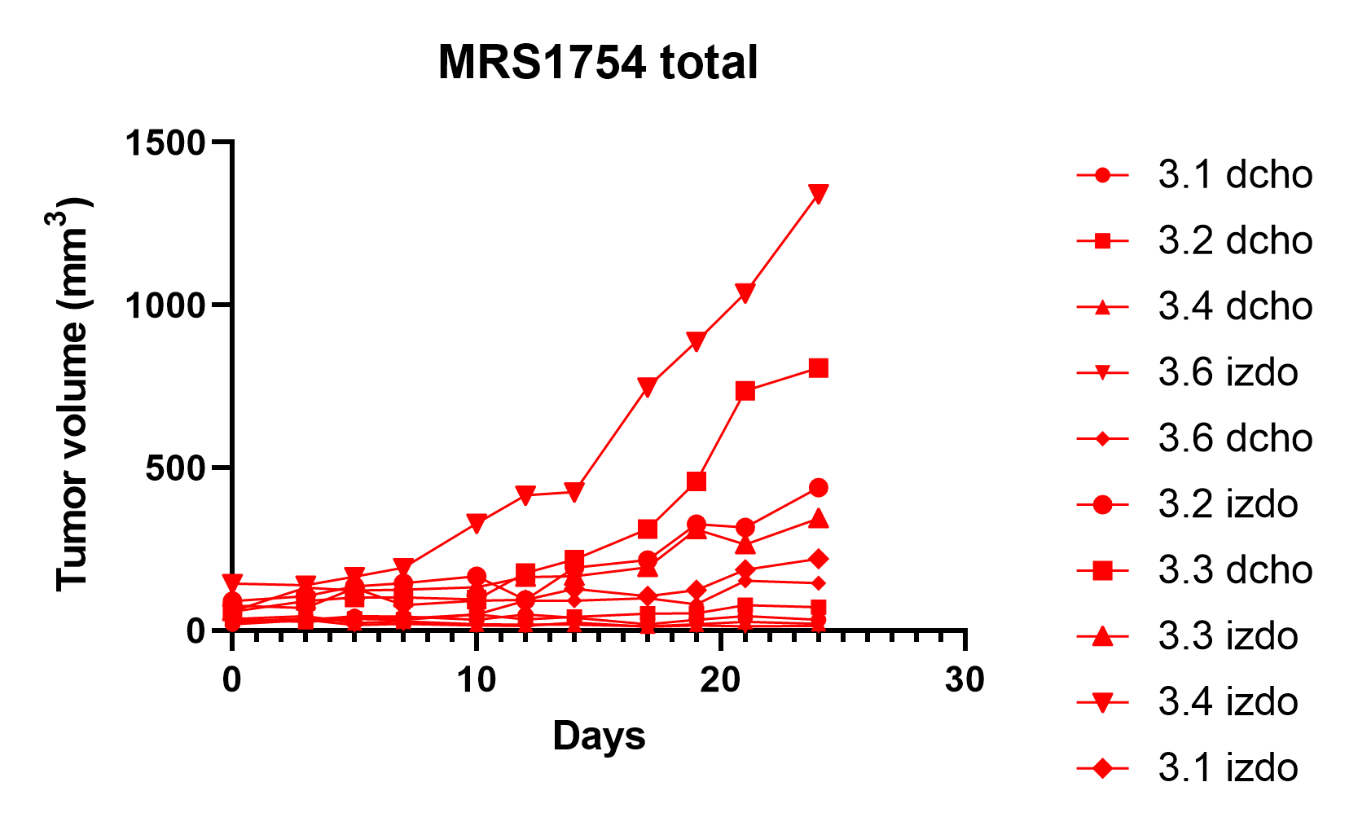


A

B


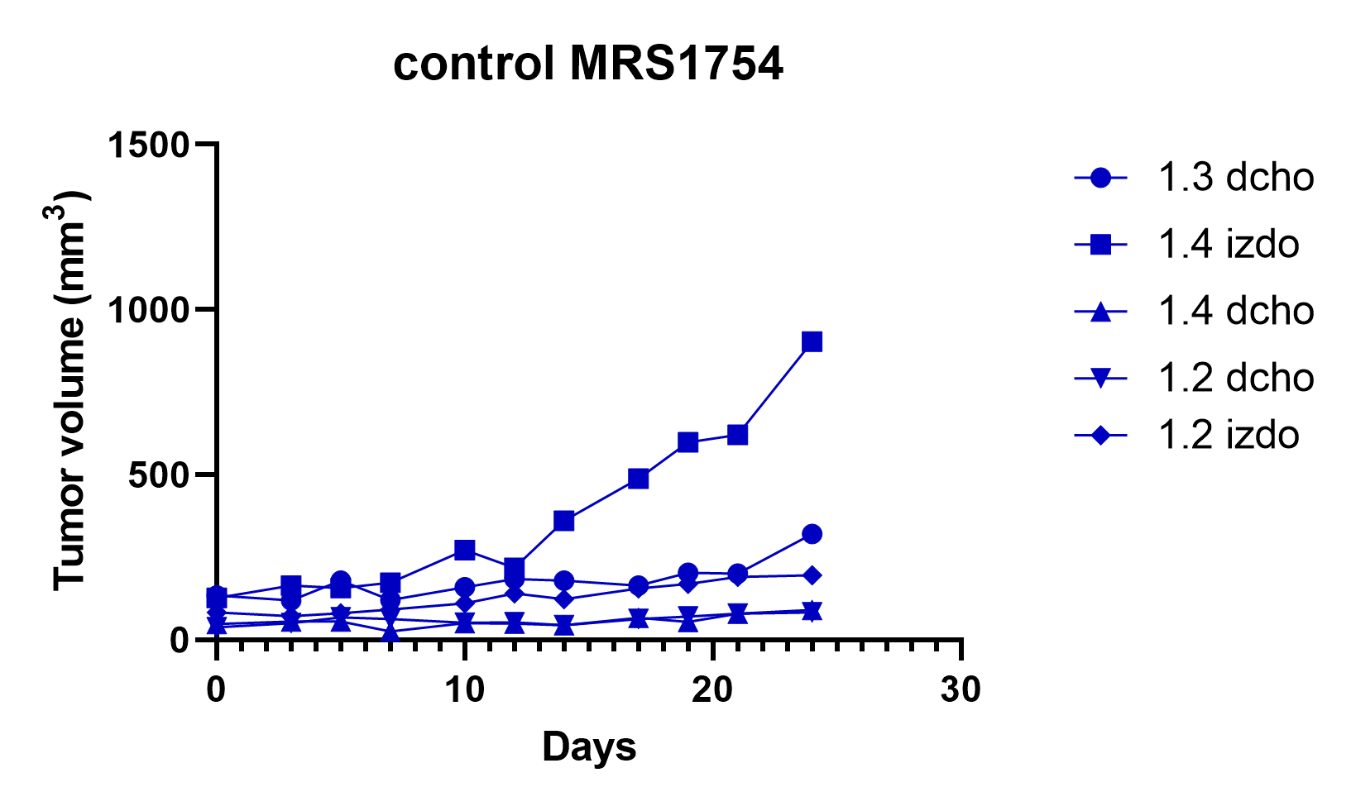


**Supplementary Figure 1**. Tumor volume is not affected by A_2B_AR pharmacologic blockade by MRS1754 in vivo. Graph of tumour size (mm^3^) of *in vivo* treatment. A) MRS1754 160ng/Kg/48 hr in 0.01% DMSO B) 1X PBS-0.001% DMSO (Vehicle)


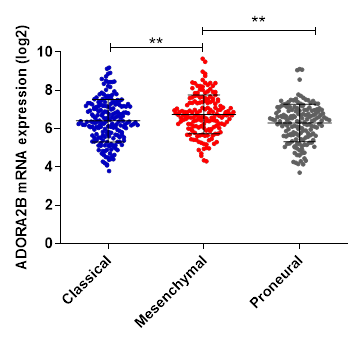


**Supplementary figure 2.  A_2B_AR expression is highest in the mesenchymal subtype of GBM.**one-way ANOVA”, *** p-valor < 0,05
